# Tumour stage, treatment, and survival of women with high-grade serous tubo-ovarian cancer in UKCTOCS: an exploratory analysis of a randomised controlled trial

**DOI:** 10.1016/S1470-2045(23)00335-2

**Published:** 2023-09-01

**Authors:** Usha Menon, Aleksandra Gentry-Maharaj, Matthew Burnell, Andy Ryan, Naveena Singh, Ranjit Manchanda, Jatinderpal K Kalsi, Robert Woolas, Rupali Arora, Laura Casey, Anne Dawnay, Aarti Sharma, Karin Williamson, Sophia Apostolidou, Lesley Fallowfield, Alistair J McGuire, Stuart Campbell, Steven J Skates, Ian J Jacobs, Mahesh K B Parmar

**Affiliations:** MRC Clinical Trials Unit at UCL, Institute of Clinical Trials and Methodology, University College London, London, UK; MRC Clinical Trials Unit at UCL, Institute of Clinical Trials and Methodology, University College London, London, UK; Department of Women’s Cancer, Elizabeth Garrett Anderson Institute for Women’s Health University College London, London, UK; MRC Clinical Trials Unit at UCL, Institute of Clinical Trials and Methodology, University College London, London, UK; Department of Cellular Pathology, Barts Health NHS Trust, London, UK; Department of Gynaecological Oncology Barts Health NHS Trust, London, UK; Barts Health NHS Trust, London, UK; Wolfson Institute of Population Health, CRUK Barts Cancer Centre, Queen Mary University of London, London, UK; AGE Research Unit, School of Public Health Imperial College London, London, UK; Department of Gynaecological Oncology, Queen Alexandra Hospital, Portsmouth, UK; Department of Cellular Pathology, University College London, Hospitals NHS Trust, London, UK; Department of Cellular Pathology Barts Health NHS Trust, London, UK; Department of Clinical Biochemistry Barts Health NHS Trust, London, UK; Department of Obstetrics and Gynaecology, University Hospital of Wales, Cardiff, UK; Department of Gynaecological Oncology, Nottingham University Hospitals, Nottingham, UK; MRC Clinical Trials Unit at UCL, Institute of Clinical Trials and Methodology, University College London, London, UK; Sussex Health Outcomes Research and Education in Cancer (SHORE-C), Brighton and Sussex Medical School, University of Sussex, Brighton, UK; London School of Economics and Political Science, London, UK; Create Health, London, UK; Massachusetts General Hospital and Harvard Medical School, Boston, MA, USA; Department of Women’s Cancer, Elizabeth Garrett Anderson Institute for Women’s Health University College London, London, UK; MRC Clinical Trials Unit at UCL, Institute of Clinical Trials and Methodology, University College London, London, UK

## Abstract

**Background:**

In UKCTOCS, there was a decrease in the diagnosis of advanced stage tubo-ovarian cancer but no reduction in deaths in the multimodal screening group compared with the no screening group. Therefore, we did exploratory analyses of patients with high-grade serous ovarian cancer to understand the reason for the discrepancy.

**Methods:**

UKCTOCS was a 13-centre randomised controlled trial of screening postmenopausal women from the general population, aged 50–74 years, with intact ovaries. The trial management system randomly allocated (2:1:1) eligible participants (recruited from April 17, 2001, to Sept 29, 2005) in blocks of 32 using computer generated random numbers to no screening or annual screening (multimodal screening or ultrasound screening) until Dec 31, 2011. Follow-up was through national registries until June 30, 2020. An outcome review committee, masked to randomisation group, adjudicated on ovarian cancer diagnosis, histotype, stage, and cause of death. In this study, analyses were intention-to-screen comparisons of women with high-grade serous cancer at censorship (Dec 31, 2014) in multimodal screening versus no screening, using descriptive statistics for stage and treatment endpoints, and the Versatile test for survival from randomisation. This trial is registered with the ISRCTN Registry, 22488978, and ClinicalTrials.gov, NCT00058032.

**Findings:**

202 562 eligible women were recruited (50 625 multimodal screening; 50 623 ultrasound screening; 101 314 no screening). 259 (0·5%) of 50 625 participants in the multimodal screening group and 520 (0·5%) of 101 314 in the no screening group were diagnosed with high-grade serous cancer. In the multimodal screening group compared with the no screening group, fewer were diagnosed with advanced stage disease (195 [75%] of 259 *vs* 446 [86%] of 520; p=0·0003), more had primary surgery (158 [61%] *vs* 219 [42%]; p<0·0001), more had zero residual disease following debulking surgery (119 [46%] *vs* 157 [30%]; p<0·0001), and more received treatment including both surgery and chemotherapy (192 [74%] *vs* 331 [64%]; p=0·0032). There was no difference in the first-line combination chemotherapy rate (142 [55%] *vs* 293 [56%]; p=0·69). Median follow-up from randomisation of 779 women with high-grade serous cancer in the multimodal and no screening groups was 9·51 years (IQR 6·04–13·00). At censorship (June 30, 2020), survival from randomisation was longer in women with high-grade serous cancer in the multimodal screening group than in the no screening group with absolute difference in survival of 6·9% (95% CI 0·4–13·0; p=0·042) at 18 years (21% [95% CI 15·6–26·2] *vs* 14% [95% CI 10·5–17·4]).

**Interpretation:**

To our knowledge, this is the first evidence that screening can detect high-grade serous cancer earlier and lead to improved short-term treatment outcomes compared with no screening. The potential survival benefit for women with high-grade serous cancer was small, most likely due to only modest gains in early detection and treatment improvement, and tumour biology. The cumulative results of the trial suggest that surrogate endpoints for disease-specific mortality should not currently be used in screening trials for ovarian cancer.

**Funding:**

National Institute for Health Research, Medical Research Council, Cancer Research UK, The Eve Appeal.

## Introduction

Ovarian cancer continues to be a disease that is diagnosed at an advanced stage. Although treatments have improved, less than half of women survive for 5 years after diagnosis.^1^ The case-to-fatality ratio is nearly three times that of breast cancer, making ovarian cancer the most lethal cancer for women in high-income countries. Since the mid-1980s, the premise has been that detecting the disease earlier in asymptomatic women would reduce mortality.^2^ The results of the large, multicentre, randomised, controlled UK Collaborative Trial of Ovarian Cancer Screening (UKCTOCS) showed significant downstaging of women with ovarian cancer in the multimodal screening group compared with the no screening group. Even 9 years following the end of screening, there was a 24·5% decrease in stage IV incidence and a 47% increase in stage I disease incidence. However, there was no reduction in deaths from ovarian cancer between the screening groups and the no screening group.^3,4^ The recommendation continues to be that ovarian cancer screening should not be undertaken in the general population.^5,6^

Ovarian cancer spans a heterogenous group of neoplasms of differing histology, molecular features, and prognosis. It includes non-epithelial, borderline epithelial, and invasive epithelial ovarian and tubal cancers. The invasive cancers comprise two main groups. The majority are tubo-ovarian high-grade serous cancer or type II ovarian cancer that are characterised by aggressive behaviour and rapidly progressive disease.^7^ They contribute to most of the deaths caused by ovarian cancer. Non-high-grade serous cancers, often referred to as type I cancers, tend to grow more slowly and include low-grade serous, mucinous, endometrioid, and clear cell ovarian cancer.

Given this disease heterogeneity, to understand the UKCTOCS conundrum, there is a need to explore the effects of screening on stage, treatment, and survival by histotype, particularly in the tubo-ovarian high-grade serous cancer group. We now report an exploratory analysis of incidence, stage, treatment outcomes, and survival from randomisation in women with high-grade serous cancer in the multimodal screening group compared with those in the no screening group. Data on non-high-grade serous cancer and on the ultrasound screening group of the trial are also included.

## Methods

### Study design and participants

UKCTOCS was a randomised, controlled trial of ovarian cancer screening done at 13 trial centres based at UK National Health Service (NHS) Trusts in England, Wales, and Northern Ireland. The trial was approved by the UK North West Multi-centre Research Ethics Committee (00/8/34) on June 23, 2000. All women provided written informed consent. The trial design has been previously published,^3,4,8^ and the protocol is available online.^4,8,9^

In brief, we invited 1 243 282 women from age–sex registers of 27 NHS primary care trusts adjoining the trial centres. Between April 17, 2001, and Sept 29, 2005, 202 638 women were recruited. Inclusion criteria were women aged 50–74 years with a postmenopausal status. Exclusion criteria were bilateral oophorectomy, previous ovarian or active non-ovarian malignancy, or increased familial ovarian cancer risk. Sex was initially based on NHS age–sex registers and then self-confirmed at recruitment as at least one intact ovary was an eligibility criterion. Ethnicity and other baseline characteristics were self-reported at recruitment.

### Randomisation and masking

The trial management system confirmed eligibility and women were randomly allocated (2:1:1) to no screening, multimodal screening, or ultrasound screening, using the Visual Basic NET version 7.1 randomisation statement and the Rnd function. It allocated 32 random numbers to each trial centre, of which eight were allocated to multimodal screening, eight to ultrasound screening, and the remaining 16 to no screening. We randomly allocated each successive participant within the centre to one of the numbers and subsequently randomly allocated them into a group. Investigators and participants were aware, and the outcomes committee was masked to randomisation group.

### Procedures

The two annual screening strategies tested were screening with serum CA-125 levels interpreted using a longitudinal algorithm (risk of ovarian cancer) as a primary test plus transvaginal ultrasound as a second-line test to increase specificity (multimodal screening group), and transvaginal ultrasound alone as the primary and second-line test (ultrasound screening group). Women had a median of eight annual screens (345 570 multimodal screening; 327 775 ultrasound screening) until Dec 31, 2011. In both groups, women with persistent abnormalities were assessed by a trial clinician and were further investigated within the NHS. We deemed women who had surgery or a biopsy for suspected ovarian cancer after clinical assessment as screen positive. Screen-detected cancers were those diagnosed following positive screen findings. Women were linked, using their NHS number, to national cancer and death registration data and hospital episodes administrative records. They were also sent three postal questionnaires. Follow-up continued until June 30, 2020. Women were censored for ovarian cancer diagnosis 3 years after end of screening (Dec 31, 2014) as prespecified in the primary mortality analysis.^4^ An outcome review committee, masked to randomisation group, adjudicated on ovarian cancer diagnosis (WHO 2014)^10^ histotype, stage (FIGO 2014),^11^ and cause of death. Treatment details were extracted from hospital records.

### Outcomes

The primary outcomes for these exploratory analyses were rates of advanced stage (III, IV, or unable to stage) disease, primary surgery, and zero residual disease after debulking surgery and survival from randomisation until June 30, 2020. Secondary outcomes included rates of primary treatment with surgery and chemotherapy (which included both primary surgery with adjuvant chemotherapy and neoadjuvant chemotherapy with interval debulking surgery) and first-line combination chemotherapy, cumulative cancer incidence per 100 000 women until Dec 31, 2014, stage-specific case-fatality rates until June 30, 2020, and absolute survival differences at 10, 15, and 18 years after randomisation in women with high grade serous cancer. All outcome data was kept confidential until unmasking.

### Statistical analysis

The main hypothesis of the trial was that screening would decrease deaths caused by ovarian cancer. In 2000, we estimated that a sample size of 200 000 women at a two-sided 5% significance level for a difference in relative ovarian cancer mortality of 30% would give 80% power for the comparison of no screening versus multimodal screening and no screening versus ultrasound screening. The primary outcome of mortality and all secondary outcomes, including incidence of advanced stage disease in ovarian cancer, have been previously reported.^3,4,12^

Our null hypotheses for the exploratory analyses reported in this paper were that the observed lack of mortality benefit, despite a reduction in advanced stage ovarian cancer incidence was due to no reduction in advanced stage disease, no improvement in treatment, and no survival benefit in women diagnosed with high-grade serous cancer in the multimodal screening group compared with the no screening group. For completeness, we report similar analyses for women diagnosed with non-high-grade serous cancer in the multimodal screening group compared with the no screening group. In addition, despite there being no evidence of a reduction in advanced stage disease incidence in ovarian cancer in the ultrasound screening group compared with the no screening group in our previous analyses,^3,4^ we also provide data on women diagnosed with high-grade serous cancer and non-high-grade serous cancer in the ultrasound screening group compared with the no screening group.

Women diagnosed with invasive epithelial ovarian and tubal cancer between randomisation and censorship for primary outcome (Dec 31, 2014) were included in the current analyses. Women with non-epithelial and borderline epithelial tumours were excluded. Descriptive statistics were calculated for baseline characteristics by group. Women were grouped by histology: high-grade serous cancer and non-high-grade serous cancer. High-grade serous cancer (appendix pp 2–4) was determined using grade and histology as per 2014 WHO guidelines. We included high-grade (grade 2–3) serous carcinoma, and high-grade (grade 3) endometrioid cancers. In addition, we included historically used diagnoses, carcinosarcoma, and carcinoma non-specified that are no longer represented in current guidelines.^13^ Non-high-grade serous cancer (appendix pp 5–7) included low-grade (grade 1) serous, endometrioid (grade 1–2), clear cell, mucinous, mixed, and Brenner cancers. Women with high-grade serous cancer and non-high-grade serous cancer were analysed separately. All comparisons were by intention to screen and included all those with cancer among participants randomly allocated to the group regardless of actual screening status, with the multimodal screening and ultrasound screening groups compared separately to the no screening group. For the exploratory analyses that we present, we have used a significance level of 0·05 to provide evidence of an effect.

For high-grade serous cancer and non-high-grade serous cancer, we compared proportions of women diagnosed with cancer and cumulative cancer incidence rates per 100 000 women until Dec 31, 2014, using standard Kaplan-Meier methods, on the basis of time from randomisation to diagnosis. Death from other causes, bilateral salpingo-oopherectomy, and loss to follow-up were censoring events and were assumed to be non-informative.

Descriptive statistics were calculated for high-grade serous cancer and non-high-grade serous cancer, including tabulations for each group (multimodal screening, ultrasound screening, no screening) by intention to screen and screening status (screen detected and clinically diagnosed cancers) where applicable.

We used a χ^2^ test of independence for intention-to-screen comparisons of the respective proportions with the multimodal screening and ultrasound screening groups compared separately to the no screening group. In addition, we did subgroup analysis by stage for treatment-related outcomes. We grouped women into two categories—stage IA–IB and stage IC or higher (IC–IV and unable to stage) based on differing treatment recommendations when screening was ongoing in the trial (2001–11). Patients with stage IA–IB ovarian cancer had surgery; adjuvant chemotherapy for stage IA–IB high-grade serous cancer was not routinely given at the time, with European Society for Medical Oncology (ESMO) guidelines only stating that it could be considered.^14^ Women with stage IC or higher were recommended surgery and chemotherapy and ideally combination chemotherapy that included platinum-based agents. To facilitate comparisons with the available literature, we also calculated primary surgery rates in women with stage II–IV (including those not staged) high-grade serous cancer in the no screening group.

In women with high-grade serous cancer, we calculated stage-specific case-fatality rates by group and screening status. In women with high-grade serous cancer in the multimodal screening and no screening groups, we calculated median follow-up from randomisation. We constructed Kaplan-Meier curves for survival (with 95% confidence intervals) from randomisation until June 30, 2020. We defined survival time from randomisation to date of death due to high-grade serous cancer or censorship (June 30, 2020), or sooner if the participant died from another cause or was lost to follow-up, which was assumed to be non-informative. We used the Versatile test in anticipation of non-proportional hazards to compare the no screening and multimodal screening groups, using either women with high-grade serous cancer or all randomly allocated participants as the denominator. The absolute difference in survival in women with high-grade serous cancer was calculated at 10, 15, and 18 years in the multimodal screening group compared with the no screening group. We used Stata 17.0 for all statistical analyses. This trial is registered with ISRCTN Registry, 22488978, and ClinicalTrials.gov, NCT00058032.

### Role of the funding source

The funders of the study had no role in study design, data collection, data analysis, data interpretation, or writing of the report.

## Results

**Figure 1:**
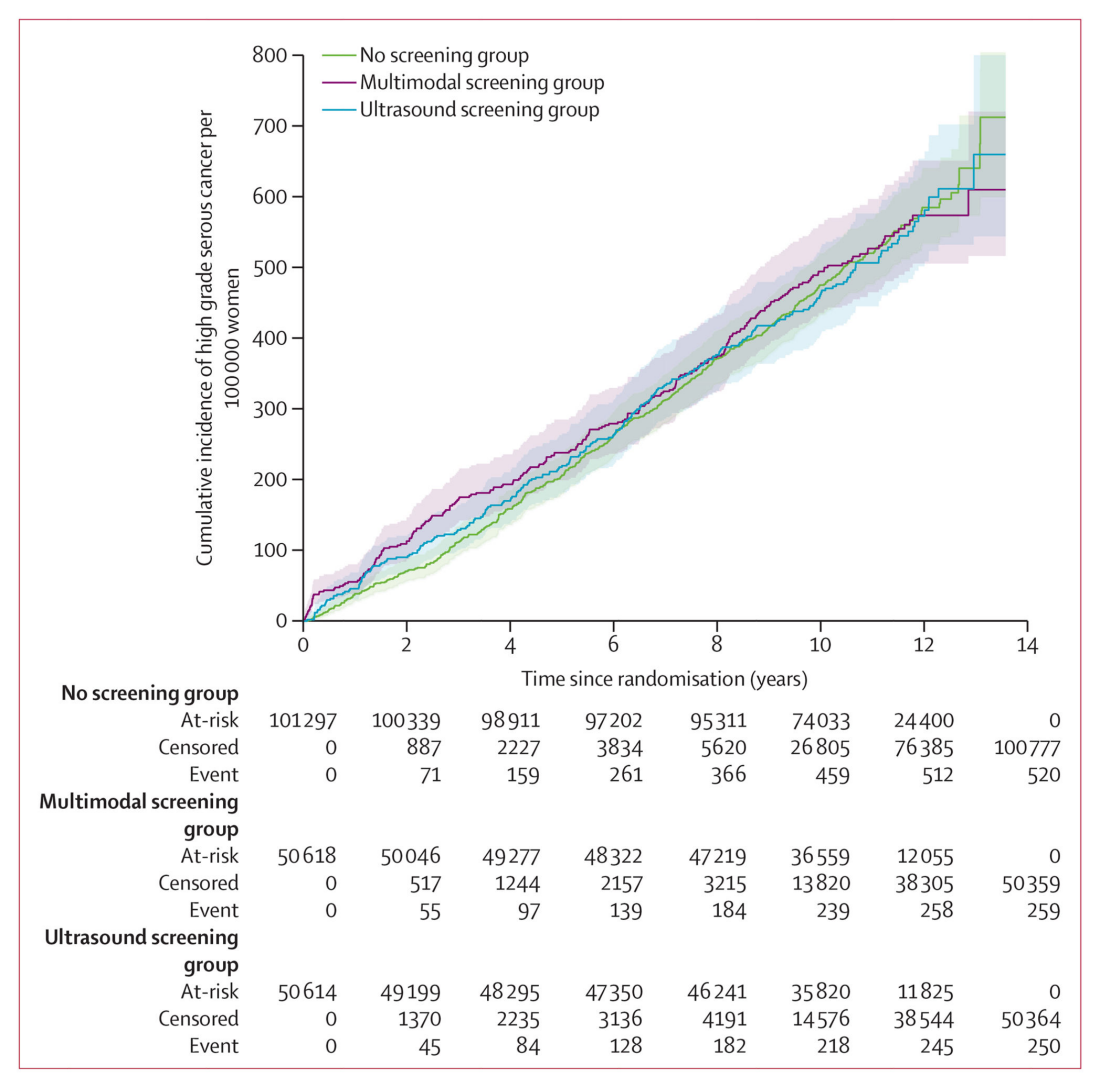
Cumulative incidence of high-grade serous tubo-ovarian cancer from randomisation until Dec 31, 2014, by screening group Shaded areas are 95% CI.

The final eligible cohort of UKCTOCS consisted of 202 562 women: 50 625 in the multimodal screening group, 50 623 in the ultrasound screening group, and 101 314 in the no screening group. Of these 202 562 participants, 1209 (0·5%) were diagnosed with invasive epithelial ovarian or tubal cancer between randomisation and primary censorship (Dec 31, 2014). 1029 (85·1%) of 1209 women had high-grade serous cancer: 259 (0·5%) of 50 625 women in the multimodal screening group, 250 (0·5%) of 50 623 in the ultrasound screening group, and 520 (0·5%) of 101 314 in the no screening group (table 1). Most cancers grouped as high-grade serous cancer (type II) were reported as high-grade serous (771 [74·9%] of 1029); historical diagnoses included in the high-grade serous cancer group were carcinoma not otherwise specified (167 [16·2%]), carcinosarcoma (53 [5·2%]), and high-grade endometrioid (38 [3·7%]). 179 (14·8%) of 1209 participants had non-high-grade serous cancer (given the small numbers, the data has not been analysed by individual histotypes): 93 no screening, 52 multimodal screening, 34 ultrasound screening, and one (<1%) had small-cell carcinoma (multimodal screening not included in the analyses). The total of 1209 includes 76 women (13 multimodal screening; 25 ultrasound screening; 38 no screening) diagnosed between randomisation and Dec 31, 2014 with missing data when we published our primary analysis.^4^ The majority of the women were White (1185 [98·0%] of 1209) and 52 (4·3%) had a previous history of breast cancer (appendix p 8). The incidence of high-grade serous cancer per 100 000 women-years was similar among the three groups: 48·0 per 100 000 women-years (95% CI 42·2–53·9; 259 cancers; 539 233 women-years) in the multimodal screening group, 47·2 per 100 000 women-years (41·4–53·1; 250 cancers; 529 531 women-years) in the ultrasound screening group, and 47·9 per 100 000 women-years (43·8–52·0; 520 cancers; 1 085 042 women-years) in the no screening group (figure 1).

Among participants diagnosed with high-grade serous cancer in the intention-to-screen population, in the multimodal screening group compared with the no screening group there was a lower diagnosis of advanced stage disease (195 [75%] of 259 *vs* 446 [86%] of 520; p=0·0003), higher rates of primary surgery (158 [61%] *vs* 219 [42%]; p<0·0001), and higher rates of zero residual disease following debulking surgery (119 [46%] *vs* 157 [30%]; p<0·0001; table 1). For women diagnosed with stage III cancer, there was no significant difference between the multimodal screening group and the no screening group for rates of zero residual disease following debulking surgery (53 [34%] of 156 with multimodal screening *vs* 84 [26%] of 325 with no screening; p=0·065). Proportions of women receiving primary treatment with surgery and chemotherapy were higher in the multimodal screening group than in the no screening group (192 [74%] *vs* 331 [64%]; p=0·003). However, there was no difference in the proportions of women receiving first line combination chemotherapy between the groups (142 [55%] *vs* 293 [56%]; p=0·69; table 1).

14 (5%) of 259 participants in the multimodal screening group were diagnosed with stage IA–IB disease compared with 14 (3%) of 520 in the no screening group (p=0·055). All women underwent primary surgery, and eight (57%) in the multimodal screening group and nine (64%) in the no screening group received adjuvant chemotherapy (p=0·71; table 1). Two (14%) received combination chemotherapy in the multimodal screening group versus three (21%) in no screening group (p=0·64; table 1).

245 (94·6%) of 259 participants in the multimodal screening group were diagnosed with stage IC or higher disease compared with 506 (97·3%) of 520 in the no screening group (p=0·055; table 1). In the subgroup of women with stage IC or higher disease, in the multimodal screening group versus the no screening group more women had primary surgery (144 [59%] *vs* 206 [41%]; p<0·0001), zero residual disease after surgery (106 [43%] *vs* 144 [28%]; p<0·0001), and primary treatment with surgery and chemotherapy (184 [74%] *vs* 322 [64%]; p=0·0062). There was no difference in the proportion of women receiving first line combination chemotherapy (140 [57%] *vs* 290 [57%]; p=1·00). The primary surgery rate in women with stage II–IV (including those not staged) high-grade serous cancer in the no screening group was 38·5% (95% CI 34·1–43·0; 187 of 486).

**Figure 2:**
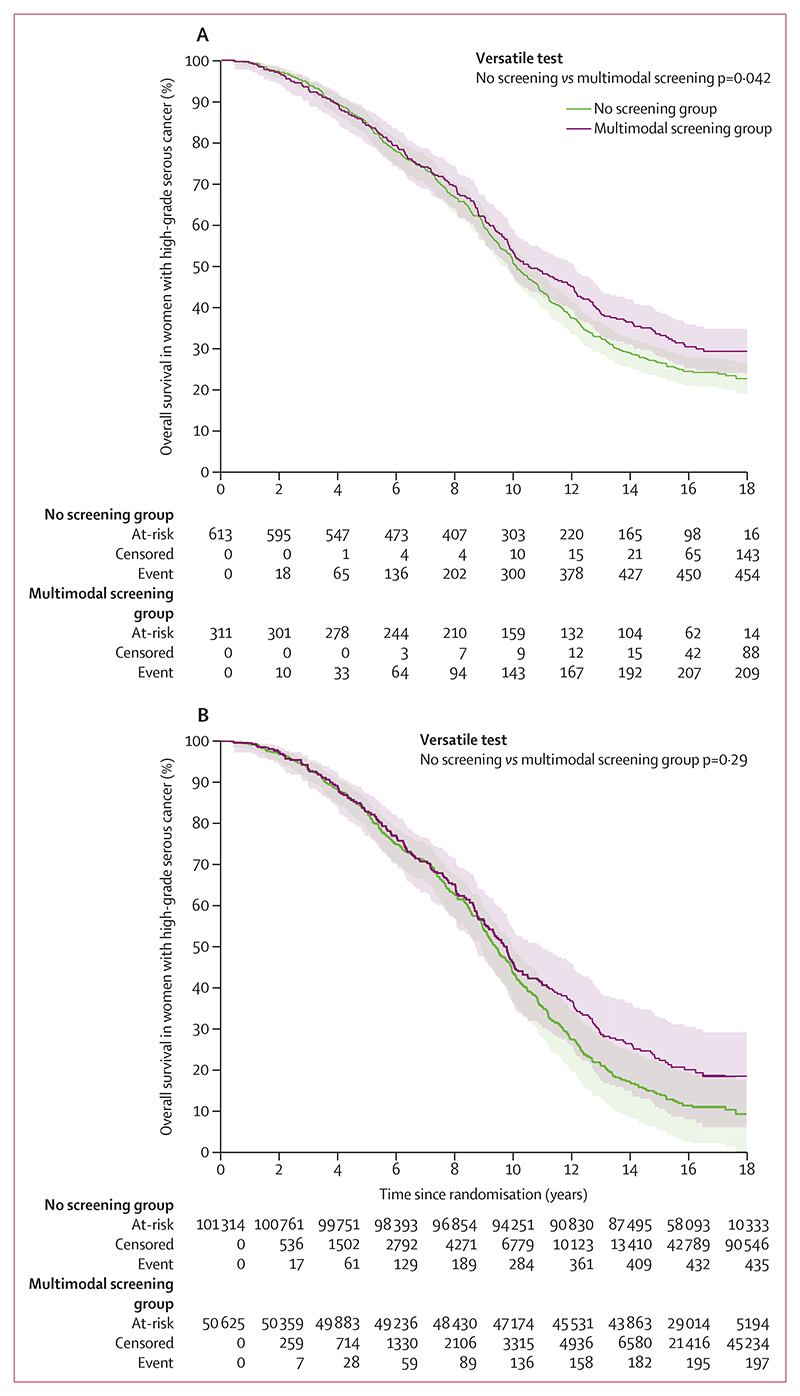
Survival from randomisation until June 30, 2020, of women with tubo-ovarian high-grade serous cancer diagnosed between randomisation and censorship (Dec 31, 2014) in the no screening and multimodal screening groups (A) Denominator is women diagnosed with high-grade serous cancer. (B) Denominator is all eligible randomised women. Shaded areas are 95% CI.

Median follow-up from randomisation in the 779 women with high-grade serous cancer in the multimodal screening (9·4; IQR 6·1–12·9) and no screening groups (9·5 years; IQR 5·1–12·6) was 9·51 years (IQR 6·04–13·00). Complete follow-up until June 30, 2020, or death date were available for 754 (97%) of 779 women (254 [95%] of 259 with multimodal screening; 508 [98%] of 520 with no screening). 205 (79%) of 259 women in the multimodal screening group and 446 (86%) of 520 in the no screening group died due to high-grade serous cancer. The case-fatality rate by stage was similar between the groups (table 2). The Versatile test showed difference (p=0·042) in overall survival from randomisation between the groups (case only survival analysis; figure 2A). The curves showed a delayed overall survival benefit in women with high-grade serous cancer in the multimodal screening group, with no difference until 10 years after randomisation (figure 2A). 5-year overall survival was 83% (95% CI 77·4 to 86·7) in the multimodal screening group versus 83% (79·4 to 85·9) in the no screening group (absolute difference –0·3%, 95% CI –5·6 to 5·3). 10-year overall survival was 46% (95% CI 40·2 to 52·4) versus 45% (40·6–49·2; absolute difference 1·5%, 95% CI –5·9 to 9·0). 15-year overall survival was 24% (95% CI 19·1 to 29·7) versus 18% (14·3 to 21·0) in the no screening group (absolute difference 6·7%, 95% CI 0·40 to 13·0). 18-year survival was 21% (95% CI 15·6 to 26·2) versus 14% (10·5–17·4; absolute difference 6·9%, 95% CI 0·61 to 13·2). When the analysis was repeated using all women randomised as the denominator, there was no difference between the multimodal screening group and no screening group (figure 2B).

The cumulative incidence of non-high-grade serous cancer in the multimodal screening group (9·6 per 100 000 women-years) was similar to that in the no screening group (8·6 per 100 000 women-years; appendix p 9). There was no difference in advanced stage disease diagnosis or treatment related endpoints in the multimodal screening group compared with the no screening group (table 3). As of June 30, 2020, 12 (23·1%) of 52 women had died due to non-high-grade serous cancer in the multimodal screening group versus 19 (20·4%) of 93 in the no screening group. No differences were observed in any of the above comparisons between the ultrasound screening group and the no screening group (tables 1, 2).

## Discussion

To our knowledge, this report provides the first evidence that screening can detect high-grade serous cancer earlier than no screening and result in improved short-term treatment outcomes. Our findings also provide evidence that the previously reported reduction in diagnosis of advanced stage disease in women with ovarian cancer in the multimodal screening group of the UKCTOCS trial occurred predominantly in those with tubo-ovarian high-grade serous cancer. This downstaging was accompanied by higher rates of primary surgery, zero residual disease after debulking surgery, and primary treatment involving surgery and chemotherapy in women with high-grade serous cancer in the multimodal screening group compared with the no screening group in an intention-to-screen analysis. However, there was no difference between the multimodal screening group and no screening group in the proportions of women with high-grade serous cancer receiving first line combination chemotherapy.

In the case-only survival analysis, there was evidence of some improvement in survival in women with high-grade serous cancer in the multimodal screening group compared with the no screening group, with an absolute difference of 6·9% at 18 years from randomisation. This survival difference was not observed when the denominator was all women who were randomly assigned. This could reflect normal variance. The case-only analysis assumes that the cancers in both groups were similar in all aspects. This assumption is supported by the similar incidence of high-grade serous cancer in both groups, which suggests that there was no screening-related overdiagnosis in the multimodal screening group. Additionally, ascertainment bias was minimised through linkage to national registers and 95% complete follow-up rates across the groups. However, we cannot exclude lead time bias entirely. There is growing evidence that high-grade serous cancer has molecular subtypes with varying survival outcomes.^15^ We do not have data on the distribution of these subtypes in the multimodal screening group and no screening group.

Our data shows that for high-grade serous cancer, downstaging alone does not capture the extent of earlier detection. Routinely available parameters, such as rates of primary surgery and zero residual disease that are important clinical outcomes,^16^ provide additional insights to lower tumour burden. It is important to consider including such parameters alongside assessment of downstaging, as intermediate endpoints in future ovarian cancer screening trials.

In keeping with the literature, the majority of the women with invasive epithelial disease had high-grade serous cancer. The similar high-grade serous cancer and non-high-grade serous cancer incidence rates in the no screening and multimodal screening groups provide strong evidence that screening did not lead to overdiagnosis in the screening group. This sets UKCTOCS apart from some previous screening trials,^17,18^ which also reported increased detection of early-stage disease but no reduction in disease-specific mortality. In these previous screening trials, unlike in UKCTOCS, there was a significant increase in cancer incidence, suggesting overdiagnosis of indolent disease in the screening groups.^17,18^

The women with high-grade serous cancer were diagnosed between 2001 and 2014. Of them, 118 (23%) of 520 in the no screening group and 26 (25%) of 106 in the clinically diagnosed multimodal screening subgroup were detected with stage IV disease. These proportions are similar to the reported stage IV disease rates for England, UK, of 21% for ovarian cancer excluding borderline neoplasms in 2012–13,^19^ and 23% for invasive serous cancers in 2016–18.^20^ It further supports the lack of ascertainment bias in the trial.

Overall, there was an 11% lower diagnosis of advanced stage high-grade serous cancer in the multimodal screening group. Larger reductions have been observed in screening trials of other cancers, such as breast, colorectal, and lung.^21^ These differences in advanced stage reductions might in part be explained by the emerging models of metastatic progression. Cancers might meta-stasise as a function of time or tumour size, or specific cell of origin and mutational lineage.^22^ In cancers with time-dependent metastasis, population-wide early detection measures present an ideal opportunity to reduce advanced disease. However, if there is a parallel progression model with metastasis occurring early and distinct metastatic clones convergently evolving, achieving large reductions in advanced stage disease might be more challenging. In high grade serous tubo-ovaran cancer, cells from premalignant serous tubal intraepithelial cancers, and perhaps even serous proliferative lesions, such as p53 signatures,^23^ can exfoliate and undergo malignant transformation in the peritoneal cavity. This parallel progression model with early metastasis suggests that achieving large reductions in stage III disease is unlikely with a screening test that only detects invasive disease. Early detection efforts are now underway to identify potential biomarkers for serous tubal intraepithelial cancer lesions.^24^ Mathematical models and evolutionary analyses suggest a 6–7 year window for a serous tubal intraepithelial cancer lesion to develop into an invasive cancer, with metastases following rapidly thereafter.^25,26^ A serous tubal intraepithelial cancer biomarker with high specificity is likely to change the screening landscape for tubo-ovarian cancer.

More frequent screening might lead to further reductions in advanced stage disease. In the UK Familial Ovarian Cancer Screening Study,^27^ women at increased risk of ovarian cancer had larger reduction in advanced stage diagnoses during 4-monthly screening compared with the follow-up period after the end of screening. During screening, 9 (47%) of 19 participants were diagnosed with advanced stage disease compared with 17 (94%) of 18 diagnosed after the end of screening, during follow-up.^27^ However, it is unlikely that women in the general population would be willing to have such intensive screening. The absolute number of false positives and the effect on resources would also be higher.

The primary surgery rates in women with stage II–IV (including those not staged) high-grade serous cancer in the no screening group was 38·5% (95% CI 34·1–43·0; 187 of 486). The rates were higher than the 29·2% reported for stage II–IV serous cancers diagnosed in 2016–18 in the national audit for England.^20^ These higher rates a decade earlier in the no screening group of UKCTOCS bear testimony to the quality of patient management within the trial. It probably reflects that fact that the 13 UKCTOCS regional trial centres were established gynaecological oncology centres.^28^

There was no difference between the multimodal screening group and no screening group in the proportions of women receiving first line combination chemotherapy, usually a platinum and a taxol. This suggests that the gains in surgical treatment were not accompanied by more women in the multimodal screening group receiving the ideal systemic treatment. This is likely to have contributed to the lack of a mortality benefit in the multimodal screening group compared with the no screening group. Of note, a higher proportion of women were diagnosed with stage IA–IB high-grade serous cancer in the multimodal screening group than in the no screening group. However, a lower proportion received combination chemotherapy in the multimodal screening group than in the no screening group. During the trial, the use of adjuvant chemotherapy for stage IA–IB high-grade serous cancer was controversial, with ESMO guidelines only stating that it could be considered.^14^ It was only in 2013, after screening had ended, that a Cochrane meta-analysis^29^ led to the guidelines^30^ recommending adjuvant chemo-therapy for all women with early-stage high-grade serous cancer. This suggests that survival differences seen in UKCTOCS could have been improved by standardising treatment of screen-detected cancers. As a standard, treatment protocols are not part of the design of screening trials because it confounds interpretation of the results and creates uncertainty as to whether early detection or treatment optimisation led to mortality reduction. However, it is likely that the aggressive cancers detected earlier through screening require a different treatment approach from clinically diagnosed early-stage cancers. The issue of treatment needs to be considered carefully and perhaps incorporated into future screening trial protocols, especially those using circulating tumour DNA-based approaches.

Key strengths of our study have been previously detailed^4^ and include scale; multicentre design; adherence to protocol through use of a bespoke, web-based trial management system with automation of key processes, remote data entry, and concurrent central monitoring; high-quality patient management in all groups of the trial; completeness of follow-up through linkage to national registries, and administrative databases; and independent adjudication of cancer site and cause of death. Follow-up until June 30, 2020, ensured completeness of data and inclusion of women with delayed registrations of ovarian cancer before censorship on Dec 31, 2014. We restaged all cases using the FIGO 2014 criteria and revised our ovarian and tubal cancer site assignment using revised WHO classification to reflect the current understanding of disease biology.

A key limitation of our study was that most women who were diagnosed with screen-detected cancer were diagnosed and treated more than a decade ago (2001–2011) and did not have the advantage of more recent advances in clinical management (eg, widespread use of ultraradical surgery, earlier treatment modulation based on better prognostic indicators, and targeted therapies) that could have improved outcomes. However, it needs to be noted that most of the advances have resulted in improvements in progression-free survival and the effect on overall survival has been modest.

Although general population screening for ovarian cancer cannot be recommended, our findings suggest that future technologies able to detect more women with high-grade serous cancer earlier, coupled with treatment improvements, might have a mortality benefit in the future. Our findings are likely to be invaluable for modeling ovarian cancer screening. The cumulative results of the trial suggest that surrogate endpoints for disease-specific mortality, such as advanced stage or better treatment outcomes, should not currently be used in place of disease-specific mortality in ovarian cancer screening trials.

## Supplementary Material

Supplementary Material

## Figures and Tables

**Table 1:** Summary of stage and treatment of women with high-grade serous tubo-ovarian cancer diagnosed between randomisation and Dec 31, 2014

	No screening group (clinically diagnosed)	Multimodal screening group					Ultrasound screening group			
		Screen detected	Clinically diagnosed	Total	p value*		Screen detected	Clinically diagnosed	Total	p value*
Randomly assigned and eligible women	101314	··	··	50 625	··		··	··	50623	··
Randomly assigned women who developed high-grade serous cancer by intention to screen	520/101314 (0-51%)	··	··	259/50 625 (0·51%)	1·00		··	··	250/50 623 (0·49%)	0·60
Cancers by screening status	520	153	106	··	··		81	169	··	··
Advanced stage by screening status †	446/520 (86%)	107/153 (70%)	88/106 (83%)	··	··		60/81 (74%)	154/169 (91%)	··	··
Advanced stage by intention to screen†	446/520 (86%)	··	··	195/259 (75%)	0·0003				214/250 (86%)	0·95
Primary surgery by screening status	219/520 (42%)	119/153 (78%)	39/106 (37%)	··	··		54/81 (67%)	50/169 (30%)	··	··
Primary surgery by intention to screen	219/520 (42%)	··	··	158/259(61%)	<0·0001		··	··	104/250 (42%)	1·00
Zero residual after surgery by screening status	157/520 (30%)	84/153 (55%)	35/106 (33%)	··	··		44/81 (54%)	39/169(23%)	··	··
Zero residual after surgery on intention to screen	157/520 (30%)			119/259 (46%)	<0·0001				83/250 (33%)	0·40
Surgery and chemotherapy by screening status	331/520 (64%)	133/153 (87%)	59/106 (56%)	··	··		66/81 (81%)	93/169 (55%)	··	··
Surgery and chemotherapy by intention to screen	331/520 (64%)	··	··	192/259 (74%)	0·003		··	··	159/250(64%)	0·97
Combination chemotherapy by screening status‡	293/520 (56%)	93/153 (61%)	49/106 (46%)	··	··		49/81 (60%)	93/169 (55%)	··	··
Combination chemotherapy by intention to screen‡	293/520 (56%)	··	··	142/259 (55%)	0·69		··	··	142/250 (57%)	0·91
Subgroup analyses										
Treatment in women with stage IA and IB	14	11	3	··	··		4	1	··	··
Surgery and chemotherapy by screening status	9/14 (64%)	6/11 (55%)	2/3 (66%)	··	··		3/4 (75%)	0	··	··
Surgery and chemotherapy by intention to screen	9/14 (64%)	··	··	8/14 (57%)	0·71		··	··	3/5 (60%)	0·88
Combination chemotherapy by screening status‡	3/14 (21%)	1/11 (9%)	1/3 (33%)	··	··		0	0	··	··
Combination chemotherapy by intention to screen‡	3/14 (21%)	··	··	2/14 (14%)	0·64		··	··	0	0·28
Treatment in women with stage IC or higher	506	142	103	··	··		77	168	··	··
Primary surgery by screening status	206/506 (41%)	108/142 (76%)	36/103 (35%)	··	··		50/77 (65%)	49/168 (29%)	··	··
Primary surgery by intention to screen	206/506 (41%)	··	··	144/245 (59%)	<0·0001		··	··	99/245 (40%)	0·94
Zero residual after surgery by screening status	144/506 (28%)	73/142 (51%)	33/103 (32%)	··	··		40/77 (52%)	38/168 (23%)	··	··
Zero residual after surgery on intention to screen	144/506 (28%)	··	··	106/245 (43%)	<0·0001		··	··	78/245 (32%)	0·26
Surgery and chemotherapy by screening status	322/506 (64%)	127/142 (89%)	57/103 (55%)	··	··		63/77(82%)	93/168 (55%)	··	··
Surgery and chemotherapy by intention to screen	322/506 (64%)	··	··	184/245 (74%)	0·006		··		156/245 (64%)	1·00
Combination chemotherapy by screening status‡	290/506 (57%)	92/142 (65%)	48/103 (47%)	··	··		49/77 (64%)	93/168 (55%)	··	··
Combination chemotherapy by intention to screen‡	290/506 (57%)	··	··	140/245 (57%)	1·00		··	··	142/245 (58%)	0·80

Data are n or n/N (%).

* All comparisons are by intention to screen between the screening group (multimodal or ultrasound) and the no screening group.

† FIGO 2014 cancer stages III, IV, or unable to stage.

‡ Combination chemotherapy includes trial drugs; majority of patients received platinum and taxol.

**Table 2:** Case fatality rates by stage on June 30, 2020, in women with high-grade serous tubo-ovarian cancer diagnosed between randomisation and Dec 31, 2014

	No screening group (clinically diagnosed)	Multimodal screening group				Ultrasound screening group		
		Screen detected	Clinically diagnosed	Total		Screen detected	Clinically diagnosed	Total
FIGO 2014 Stage								
I	16/34 (47%)	13/27 (48%)	5/10 (50%)	··		2/11 (18%)	4/7 (57%)	··
II	22/40 (55%)	9/19 (47%)	5/8 (63%)	··		4/10 (40%)	2/8 (25%)	··
III	294/325 (90%)	86/95 (91%)	53/61 (87%)	··		48/53 (91%)	92/102 (90%)	··
IV	111/118 (94%)	12/12 (100%)	21/26 (81%)	··		7/7 (100%)	48/52 (92%)	··
Unable to stage	3/3 (100%)	0	1/1 (100%)	··		0	0	··
Total by screening status	446/520 (86%)	120/153 (78%)	85/106 (80%)	··		61/81 (75%)	146/169 (86%)	··
Total by intention to screen	446/520 (86%)	··	··	205/259 (79%)		··	··	207/250 (83%)

Data are n/N (%). Median follow-up (years) from randomisation: no screening 9·5 (IQR 5·1–12·6); multimodal screening –9·7 (IQR 6·2–14·1); and ultrasound screening 9·4 (IQR 6·1–12·9).

**Table 3:** Summary of stage and treatment of women with non-high-grade serous epithelial ovarian cancer diagnosed between randomisation and Dec 31, 2014

	No screening group (clinically diagnosed)	Multimodal screening group					Ultrasound screening group			
		Screen detected	Clinically diagnosed	Total	p value*		Screen detected	Clinically diagnosed	Total	p value*
Randomly assigned and eligible women	101314	··	··	50 625	··		··	··	50 623	··
Randomly assigned women who developed non-high-grade serous cancer by intention to screen	93/101314 (<1%)			52/50 625 (<1%)	0·55				34/50 623 (<1%)	0·20
Cancers by screening status	93	27	25	··	··		24	10	··	··
Advanced stage by screening status†	19/93 (20%)	4/27 (15%)	3/25 (12%)	··	··		4/24 (17%)	5/10 (50%)	··	··
Advanced stage by Intention to screen†	19/93 (20%)	··	··	7/52 (13%)	0·29		··	··	9/34 (26%)	0·47
Primary surgery by screening status	88/93 (95%)	27/27 (100%)	24/25 (96%)	··	··		23/24 (96%)	8/10 (80%)	··	··
Primary surgery by intention to screen	88/93 (95%)	··	··	51/52 (98%)	0·37		··	··	31/34 (91%)	0·41
Zero residual after surgery by screening status	80/93 (86%)	24/27 (89%)	21/25 (84%)	··	··		20/24 (83%)	7/10 (70%)	··	··
Zero residual after surgery on intention to screen	80/93 (86%)	··	··	45/52 (87%)	0·87		··	··	27/34 (79%)	0·34
Surgery and chemotherapy by screening status	66/93 (71%)	17/27 (63%)	15/25 (60%)	··	··		18/24 (75%)	4/10 (40%)	··	··
Surgery and chemotherapy by intention to screen	66/93 (71%)	··	··	32/52 (62%)	0·27		··	··	22/34 (65%)	0·52
Combination chemotherapy by screening status*	34/93 (37%)	7/27 (26%)	9/25 (36%)	··	··		10/24 (42%)	1/10 (10%)	··	··
Combination chemotherapy by intention to screen*	34/93 (37%)	··	··	16/52 (31%)	0·48		··	··	11/34 (32%)	0·66
Subgroup analyses										
Treatment in women with stage IA and IB	24	9	10				7	4		··
Surgery and chemotherapy by screening status	8/24 (33%)	3/9 (33%)	2/10 (20%)				2/7 (29%)	0		··
Surgery and chemotherapy by intention to screen	8/24 (33%)			5/19 (26%)	0·62			··	2/11 (18%)	0·37
Combination chemotherapy by screening status‡	3/24 (13%)	2/9 (22%)	1/10 (10%)				0	0		··
Combination chemotherapy by intention to screen‡	3/24 (13%)			3/19 (16%)	0·76				0	0·22
Treatment in women with stage IC or higher§	69	18	15				17	6		··
Primary surgery by screening status	64/69 (93%)	18/18 (100%)	14/15 (93%)				16/17 (70%)	4/6 (67%)		··
Primary surgery by intention to screen	64/69 (93%)			32/33 (97%)	0·40				20/23 (87%)	0·39
Zero residual after surgery by screening status	56/69 (81%)	15/18 (83%)	11/15 (73%)				13/17 (57%)	3/6 (50%)		··
Zero residual after surgery on intention to screen	56/69 (81%)			26/33 (79%)	0·78				16/23 (70%)	0·24
Surgery and chemotherapy by screening status	58/69 (84%)	14/18 (78%)	13/15 (87%)				16/17 (70%)	4/6 (67%)		··
Surgery and chemotherapy by intention to screen	58/69 (84%)			27/33 (82%)	0·78				20/23 (87%)	0·74
Combination chemotherapy by screening status‡	31/69 (45%)	5/18 (28%)	8/15 (53%)				10/17 (59%)	1/6 (17%)		··
Combination chemotherapy by intention to screen‡	31/69 (45%)			13/33 (39%)	0·60				11/23 (48%)	0·81

Data are n or n/N (%).

* All comparisons are intention to screen between the screening group (multimodal or ultrasound) and the no screening group.

† FIGO 2014 cancer stages III, IV, or unable to stage.

‡ Combination chemotherapy includes trial drugs; majority of patients received platinum and taxol.

§ Stage IC–IV and unable to stage.

## Data Availability

The protocol is available on the study website. The individual participant data that underlie the results reported in this Article, after de-identification, will be available beginning 12 months after publication. A data dictionary defining each field in the set will be made available. Researchers will need to state the aims of any analyses and provide a methodologically sound proposal. Proposals should be directed to u.menon@ucl.ac.uk. Data requestors will need to sign a data access agreement and in keeping with patient consent for secondary use, obtain ethical approval for any new analyses. Following all necessary approvals and mandatory training required for access to UKCTOCS data, the researchers will be given access to the data, which is housed within the UCL Data Safe Haven.

## Research in context

### Evidence before this study

We searched PubMed for publications with no language restrictions from Jan 1, 2015, to Jan 1, 2023, using search terms “ovarian cancer” AND “screening” AND “randomised controlled trial” AND “mortality” to identify relevant publications. We found two screening trials that have reported on ovarian cancer mortality. The US Ovarian Cancer Screening group of the PLCO Cancer Screening trial included 78 216 postmenopausal women. The trial reported no reduction in advanced stage disease nor a mortality benefit with screening, either at the initial follow-up (median 12·4 years) or the long-term follow-up (median 14·7 years). The largest randomised, controlled trial on ovarian cancer screening, UKCTOCS, included 202 638 postmenopausal women. Annual screening compared with no screening showed a significant reduction in the diagnosis of advanced stage ovarian cancer with multimodal screening but not with ultrasound screening, both at initial follow-up (median 11·1 years) and long-term follow-up (median 16·3 years). However, there was no reduction in disease-specific mortality. There were no data available on stage, treatment, or mortality of women with high-grade serous tubo-ovarian cancer in either of the trials.

### Added value of this study

This exploratory study details stage, treatment, and survival of women with high-grade serous tubo-ovarian cancer diagnosed between randomisation and Dec 31, 2014, in UKCTOCS. The findings provide evidence that the previously reported reduction in the diagnosis of advanced stage ovarian cancer in the multimodal screening group compared with the no screening group occurred predominantly in those with high-grade serous cancer. The downstaging in women with high-grade serous cancer was accompanied by higher rates of primary surgery, zero residual disease after debulking surgery, and primary treatment involving surgery and chemotherapy in an intention-to-screen analysis. However, there was no difference between groups in first line combination chemotherapy rates. In a high-grade serous cancer case-only analysis, there was a small improvement in survival from randomisation in the multimodal screening group compared with the no screening group.

### Implications of all the available evidence

At present, general population screening for ovarian cancer cannot be recommended because there was no mortality benefit in UKCTOCS. However, to our knowledge, the trial provides the first evidence that screening can detect high-grade serous tubo-ovarian cancer earlier than no screening and improve short-term treatment outcomes. The potential survival benefit was small, most likely due to modest gains in early detection and treatment improvement. This suggests that newer technologies that can detect more women with high-grade serous cancer earlier, coupled with treatment improvements and a better understanding of tumour biology, are likely to achieve a mortality benefit. The cumulative results of the trial findings suggest that surrogate endpoints for disease-specific mortality are currently unreliable in ovarian cancer screening trials.
