# Supplementary material for: Tumour stage, treatment, and survival of women with high-grade serous tubo-ovarian cancer in UKCTOCS: an exploratory analysis of a randomised controlled trial
Source: Lancet Oncol. Author manuscript; Available in PMC 2024 Nov 18. (PMC7616829; doi:10.1016/S1470-2045(23)00335-2)
Supplement: Supplementary Material [file EMS200031-supplement-Supplementary_Material.pdf]

THE LANCET
Oncology

Supplementary appendix

This appendix formed part of the original submission and has been peer reviewed. We post it as supplied by the authors.

Supplement to: Menon U, Gentry-Maharaj A, Burnell M, et al. Tumour stage, treatment, and survival of women with high-grade serous tubo-ovarian cancer in UKCTOCS: an exploratory analysis of a randomised controlled trial. *Lancet Oncol* 2023; **24:** 1018–28.

Contents

A. Web Table 1: Morphology, stage and treatment of high grade serous tubo-ovarian cancer (HGSC) diagnosed between randomisation and 31 December 2014 2

B. Web Table 2: Morphology, stage and treatment of non-high grade serous epithelial ovarian cancer (non HGSC) diagnosed between randomisation and 31 December 2014 5

C. Web Table 3: Baseline characteristics of women with tubo-ovarian cancer diagnosed between randomisation and 31 December 2014 8

D. Web Figure 1: Cumulative incidence (95% confidence bands) of nonhigh grade serous ovarian cancer (non-HGSC) from randomisation till 31 December 2014 by randomisation group 9

E. UKCTOCS committees and teams 10

A. Web Table 1: Morphology, stage and treatment of high grade serous tubo-ovarian cancer (HGSC) diagnosed between randomisation and 31 December 2014

	No screening (C) group (101314 women)	Multimodal (MMS) group (50625 women)		Ultrasound (USS) group (50623 women)	
**Characteristic, No. (% of cases)**	**C: Clinically diagnosed 520 (100)**	**MMS: Screen detected 153 (100)**	**MMS: Clinically diagnosed 106 (100)**	**USS: Screen detected 81 (100)**	**USS: Clinically diagnosed 169 (100)**
**Morphology**					
Serous, high grade	384 (74)	123 (80)	74 (70)	65 (80)	125 (74)
Carcinoma Nonspecific	89 (17)	15 (10)	23 (22)	7 (9)	33 (20)
Carcinosarcoma	34 (7)	3 (2)	4 (4)	3 (4)	9 (5)
Endometrioid, high grade	13 (3)	12 (8)	5 (5)	6 (7)	2 (1)
**FIGO 2014 Stage**					
Stage I	34 (7)	27 (18)	10 (9)	11 (14)	7 (4)
Stage II	40 (8)	19 (12)	8 (8)	10 (12)	8 (5)
Stage III	325 (63)	95 (62)	61 (58)	53 (65)	102 (60)
Stage IV	118 (23)	12 (8)	26 (25)	7 (9)	52 (31)
Unable to stage	3 (1)		1 (1)		
Advanced stage (III/IV/unable to stage) by screening status	446 (86)	107 (70)	88 (83)	60 (74)	154 (91)
Advanced stage (III/IV/unable to stage) on Intention to screen*	446 (86)	195 (75 of 259)		214 (86 of 250)	
**Treatment**					
Stage Ia and Ib	**14 (100)**	**11 (100)**	**3 (100)**	**4 (100)**	**1 (100)**
Primary surgery with adjuvant chemotherapy	9 (64)	6 (55)	2** (66)	3 (75)	
Primary surgery but no chemotherapy	4 (29)	5 (45)	1 (33)	1 (25)	1 (100)
Chemotherapy but no surgery	1*** (7)				
Stage Ic and greater including unable to stage	**506 (100)**	**142 (100)**	**103 (100)**	**77 (100)**	**168 (100)**
Primary surgery with adjuvant chemotherapy	192 (38)	108 (76)	33 (32)	47 (61)	45 (27)

Neoadjuvant chemotherapy with interval debulking surgery	130	(26)	19	(13)	24	(23)	16	(21)	48	(29)
Surgery but no chemotherapy	14	(3)			3	(3)	2	(3)	4	(2)
Chemotherapy but no surgery	129	(25)	13	(9)	23	(22)	11	(14)	53	(32)
No surgery or chemotherapy	41	(8)	2	(1)	20	(19)			18	(11)
Surgery data present but chemo data missing							1	(1)		
Women treated with surgery and chemotherapy by screening status	322	(64)	127	(89)	57	(55)	63	(82)	93	(55)
Women treated with surgery and chemotherapy on Intention to screen*	322	(64)	184	(75 of 259)			156	(64 of 250)		
Women treated with primary surgery by screening status	206	(41)	108	(76)	36	(35)	50	(65)	49	(29)
Women treated with primary surgery on Intention to screen*	206	(41)	144	(59 of 259)			99	(40 of 250)		
**1st line chemotherapy agent**										
Stage Ia and Ib	**14**	**(100)**	**11**	**(100)**	**3**	**(100)**	**4**	**(100)**	**1**	**(100)**
Combination chemotherapy****	3	(21)	1	(9)	1	(33)				
Single agent- platinum	7	(50)	5	(45)	1	(33)	2	(50)		
Missing							1	(25)		
Not Applicable	4	(29)	5	(45)	1	(33)	1	(25)	1	(100)
Women treated with combination chemotherapy by intention to screen	3	(21)	2	(14 of 14)			0	(0 of 5)		
Stage Ic and greater including unable to stage	**506**	**(100)**	**142**	**(100)**	**103**	**(100)**	**77**	**(100)**	**168**	**(100)**
Combination chemotherapy****	290	(57)	92	(65)	48	(47)	49	(64)	93	(55)
Single agent- platinum	149	(29)	45	(32)	30	(29)	22	(29)	48	(29)
Missing	11	(2)	3	(2)	2	(2)	4	(5)	5	(3)
Not Applicable	56	(11)	2	(1)	23	(22)	2	(3)	22	(13)
Women treated with combination chemotherapy by intention to screen	290	(57)	140	(57 of 245)			142	(58 of 245)		
**Residual disease after surgery**										
0 mm	157	(30)	84	(55)	35	(33)	44	(54)	39	(23)
>0 mm - 10 mm	99	(19)	42	(27)	13	(12)	15	(19)	37	(22)
>= 10 mm	87	(17)	12	(8)	12	(11)	9	(11)	19	(11)
Missing	6	(1)			3	(3)	2	(2)	3	(2)

Not applicable	171 (33)	15 (10)	43 (41)	11 (14)	71 (42)
Women with zero residual on Intention to screen*	157 (30)	119 (46 of 259)		83 (33 of 250)	

NOTE. **For intention to screen analysis, screen detected and clinically diagnosed are combined for each screening group. ** Includes one woman who had completion surgery after chemotherapy. *** Prior to debulking surgery this woman suffered a stroke and deteriorated rapidly. ****Majority platinum and taxol, includes trial drugs*

Abbreviations: *HGSC, high grade serous ovarian carcinoma; MMS, multimodal screening; USS, ultrasound screening; FIGO, International Federation of Gynecology and Obstetrics.*

B. Web Table 2: Morphology, stage and treatment of non-high grade serous epithelial ovarian cancer (non HGSC) diagnosed between randomisation and 31 December 2014

Characteristics, No. (% of cases)	No screening (C) group<br>C: Clinically diagnosed		Multimodal (MMS) group<br>MMS: Screen detected		Multimodal (MMS) group<br>MMS: Clinically diagnosed		Ultrasound (USS) group<br>USS: Screen detected		Ultrasound (USS) group<br>USS: Clinically diagnosed	
	**93**	**(100)**	**27**	**(100)**	**25**	**(100)**	**24**	**(100)**	**10**	**(100)**
**Morphology**										
Endometrioid, low grade	25	(27)	10	(37)	8	(32)	5	(21)	2	(20)
Clear Cell	21	(23)	5	(19)	11	(44)	11	(46)	1	(10)
Serous, low grade	19	(20)	11	(41)	1	(4)	5	(21)	3	(30)
Mucinous	25	(27)	1	(4)	5	(20)	3	(13)	4	(40)
Mixed Cell	2	(2)								
Brenner	1	(1)								
**FIGO 2014 Stage**										
Stage I	62	(67)	20	(74)	20	(80)	17	(71)	5	(50)
Stage II	12	(13)	3	(11)	2	(8)	3	(13)		
Stage III	16	(17)	3	(11)	3	(12)	4	(17)	4	(40)
Stage IV	3	(3)	1	(4)					1	(10)
Advanced stage (III/IV/unable to stage) by screening status	19	(20)	4	(15)	3	(12)	4	(17)	5	(50)
Advanced stage (III/IV/unable to stage) on Intention to screen*	19	(20)	7 (13 of 52)				9 (26 of 34)			
**Treatment**										
Stage Ia and Ib	**24**	**(100)**	**9**	**(100)**	**10**	**(100)**	**7**	**(100)**	**4**	**(100)**
Primary surgery with adjuvant chemotherapy	8	(33)	3	(33)	2	(20)	2	(29)		
Primary surgery but no chemotherapy	16	(67)	6	(67)	8	(80)	5	(71)	4	(100)
Women treated with curative intent by intention to treat	24	(100)	19 (100 of 19)				11 (100 of11)			
Stage Ic and greater including unable to stage	**69**	**(100)**	**18**	**(100)**	**15**	**(100)**	**17**	**(100)**	**6**	**(100)**

Primary surgery with adjuvant chemotherapy	57	(83)	14	(78)	13	(87)	16	(94)	3	(50)
Neoadjuvant chemotherapy with interval debulking surgery	1	(1)							1	(17)
Primary surgery but no chemotherapy	7	(10)	4	(22)	1	(7)			1	(17)
Chemotherapy but no surgery	2	(3)			1	(7)	1	(6)		
No surgery or chemotherapy	2	(3)							1	(17)
Women treated with curative intent by screening status	58	(84)	14	(78)	13	(87)	16	(94)	4	(67)
Women treated with curative intent on Intention to screen*	58	(84)	27	(82 of 33)			20	(87 of 23)		
Women treated with primary surgery on Intention to screen*	64	(93)	32	(97 of 33)			20	(87 of 23)		
**1st line chemotherapy agent**										
Stage Ia and Ib	**24**	**(100)**	**9**	**(100)**	**10**	**(100)**	**7**	**(100)**	**4**	**(100)**
Combination chemotherapy**	3	(13)	2	(22)	1	(10)				
Single agent- platinum	5	(21)	1	(11)	1	(10)	2	(29)		
Not Applicable	16	(67)	6	(67)	8	(80)	5	(71)	4	(100)
Women treated with platinum and taxol by intention to screen	3	(13)	3	(16 of 19)			0	(0 of 11)		
Stage Ic and greater including unable to stage	**69**	**(100)**	**18**	**(100)**	**15**	**(100)**	**17**	**(100)**	**6**	**(100)**
Combination chemotherapy**	31	(45)	5	(28)	8	(53)	10	(59)	1	(17)
Single agent- platinum	28	(41)	8	(44)	6	(40)	7	(41)	3	(50)
Missing	1	(1)	1	(6)						
Not Applicable	9	(13)	4	(22)	1	(7)			2	(33)
Women treated with platinum and taxol by intention to screen	31	(45)	13	(39 of 33)			11	(48 of 23)		
**Residual disease**										
0 mm	80	(86)	24	(89)	21	(84)	20	(83)	7	(70)
>0 mm - 10 mm	6	(6)	2	(7)	1	(4)	2	(8)	1	(10)
>= 10 mm	3	(3)	1	(4)	2	(8)	1	(4)		
Missing									1	(10)
Not applicable	4	(4)			1	(4)	1	(4)		

Women with zero residual on Intention to screen*	80 (86)	45 (87 of 52)	27 (79 of 34)

NOTE. ** For intention to screen analysis, screen detected and clinically diagnosed are combined for each screening group.*
***Majority platinum and taxol, includes trial drugs.*

Abbreviations: *LGSC, low grade serous carcinoma; MMS, multimodal screening; USS, ultrasound screening; FIGO, International Federation of Gynecology and Obstetrics; ITT, intention to treat; mm, millimetres; NA, not applicable.*

C. Web Table 3: Baseline characteristics of women with tubo-ovarian cancer diagnosed between randomisation and 31 December 2014

Characteristic, n (%) or median (IQR)	No screening (C) group (613 women)	Multimodal (MMS) group (312 women)	Ultrasound (USS) group (284 women)
Age at randomisation	62.7 (57.3-67.4)	62.5 (58.3-67.5)	63.1 (58.5-68.0)
Ethnic origin			
White	600 (97.9%)	305 (97.8%	280 (98.6%)
Non-white	7 (1.1%)	3 (1.0%)	1 (0.4%)
Other	4 (0.7%)	3 (1.0%)	2 (0.7%)
Missing	2 (0.3%)	1 (0.3%)	1 (0.4%)
Hysterectomy	120 (19.6%)	59 (18.9%)	53 (17.0%)
Ever use of OCP	314 (51.3%)	143 (45.8%)	137 (43.9%)
Pregnancies <6 months	0 (0-1)	0 (0-1)	0 (0-1)
Children (pregnancies >6 months)	2 (2-3)	2 (2-3)	2 (1-3)
Personal history of breast cancer	29 (4.7%)	11 (3.5%)	12 (3.8%)
Maternal history of ovarian cancer	13 (2.1%)	6 (1.9%)	6 (1.9%)
Maternal history of breast cancer	40 (6.5%)	22 (7.1%)	24 (7.7%)
Age at ovarian cancer diagnosis	68.3 (63.7-73.3)	67.9 (63.0-72.8)	68.8 (63.2-73.9)

Abbreviations: *IQR=inter quartile range; MMS=multimodal screening. USS=ultrasound screening. OCP=oral contraceptive pill.*

D. Web Figure 1: Cumulative incidence (95% confidence bands) of nonhigh grade serous ovarian cancer (non-HGSC) from randomisation till 31 December 2014 by randomisation group

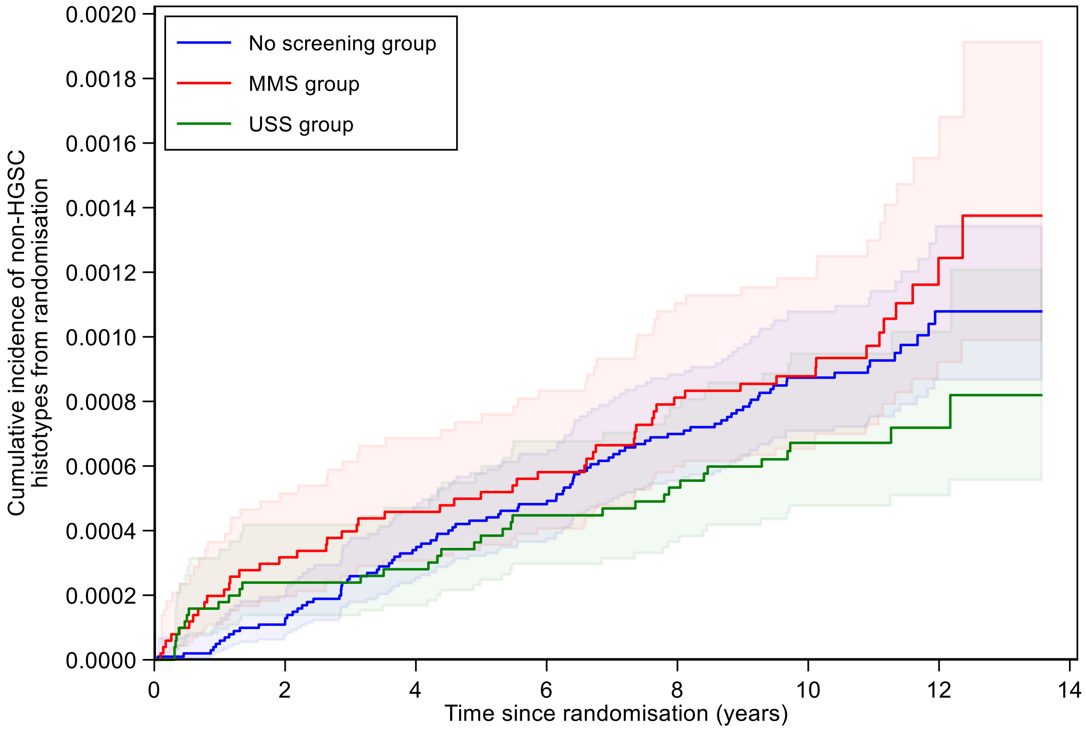

Numbers at risk

No screening	101297	(12)	100339	(22)	98911	(14)	97202	(21)	95311	(16)	74033	(8)	24400	(0)	0
MMS	50618	(16)	50046	(7)	49277	(6)	48322	(11)	47219	(3)	36559	(8)	12055	(1)	0
USS	50614	(12)	49199	(2)	48295	(8)	47350	(4)	46241	(6)	35820	(1)	11825	(1)	0

E. UKCTOCS committees and teams

Trial investigators
Prof U Menon (CI 2015-2021, co-CI 2001-2015), Prof I Jacobs (CI 2015-2021), Prof M Parmar OBE, Dr S Skates, Prof S Campbell, Prof A McGuire, Prof Dame L Fallowfield

Trial Steering Committee (2001-2021)
Prof D Luesley (Independent Chair; 2001-2015), Prof H Kitchener (Independent Chair; 2016-2021), Prof J Patnick CBE (independent member), Prof J Cuzick (independent member), Ms L Bayne (independent member; 2001-2014), Ms Annwen Jones OBE (independent member; 2017-2021), Prof U Menon, Prof I Jacobs, Prof M Parmar OBE, Prof Dame L Fallowfield

Data Monitoring and Ethics Committee (2001-2014)
Prof P Boyle (Chair), Prof APM Heintz, Prof S Kjaer, Dr Edward L Trimble

Trial Management Committee (2001-2021)
Prof U Menon (Chair 2015-2021), Prof I Jacobs (Chair 2001-2014), Prof S Campbell, Prof Dame L Fallowfield, Prof A McGuire, Prof M Parmar OBE, Dr S Skates, Mr R Woolas, Mr T Mould, Dr N Singh, Dr A Dawnay, Dr A Gentry-Maharaj, Dr J Kalsi, Dr A Ryan, Dr M Burnell

Outcome Review Committee (2001-2021)
Dr N Singh (Chair), Dr E Benjamin (2001-2014), Ms K Reynolds (2001-2005) , Prof M Widschwendter (2009-2014), Mr Rob Woolas, Prof Ranjit Manchanda (2017-2021), Mr Tim Mould (2001-2014), Ms Karin Williamson (2018-2021), Ms Aarti Sharma (2018-2021), Dr Rupali Arora (2019-2021),Dr Laura Casey (2020-2021)

Ultrasound Sub-Committee (2001-2014)
Prof U Menon (Chair), Prof N Amso, Dr C Brunell, Prof S Campbell, Mrs G Fletcher, Ms K Ford, Dr A Gentry-Maharaj, Dr J Kalsi, Ms R Rangar, Dr A Ryan, Mr M Seif, Dr G Turner

Coordinating Centre Team
Prof U Menon (Lead), M Ahmad , T Akbar, N Alves, Dr S Apostolidou, M Bacon, Dr C Brunell, Dr M Burnell, G Carlino J Chapman, D Crump, J Cunningham, L Danquah, S Davies, Dr A Dawnay, A Dyer, J Ford, Dr A Gentry-Maharaj (Trial Manager 2017-2021), A Gibson, T Goodall, S Grant, R Gunu, M Habib, L Hadcocks, Dr R Hallett, N Hinkey, Dr J Kalsi (Trial Manager 2009-2017), C Karpinskyj, J Kerkhoff, Z Khan, S Lewis, W Liston, S Mohamed, L Odunlami, M Pamboris, S Philpott, T Roberts, Dr A Ryan, Dr A Sharma, J Sheals, K Sibley, C Spicer, S Spicer, L Sterry, C Stubbs, K Tamm, J Taylor, Dr F Warburton, Y Wold, T Roberts.

Regional Trial Centre Teams (n=number randomised)
Department of Gynaecological Oncology, St. Bartholomew's Hospital, London (n=19996)
D Oram (Lead), U Menon (Lead), J Ademi, C Amarasinghe, CM Baque-Juxton, S Bhola, J Bramble, J Chapman, J Charalambous, A Clough, L Cole, L Crosby, J Cunningham, E d'Tisi, E Ferrier, E Forde, P Goulding, B Heyer, J Jonsson, A Knowles, E Liu-Koo, AM Mackinson, V Medic, U Menon, A Relf, K Reynolds, B Rufford, E Ryan, S Sheik, C Stubbs, L Walters, D Warrington, J Webb.

Department of Gynaecological Oncology, St. Mary's Hospital, Portsmouth (n=19180)
R Woolas (Lead), S Aldcock, M Anderson, E Barclay, ? Bell, R Bonner, E Bowes, D Brinkmann, J Burns, K Chorley, C Dhar, K Fairley, F Gardner, B Gibbs, Y Griffiths, R Harrison, L Hayward, C Ihezue, C Isaac, D James, F Jones, D Mason, E Merritt, R Morris, M Oakey, J Skinner, S Tilbury, J Turpitt, A Webb, C West, J Woolas.

Northern Gynaecological Oncology Centre, Queen Elizabeth Hospital, Gateshead (n= 17326)
K Godfrey (Lead), A Lopes (Lead), J Callaghan, G Dorman, J Gibson, C Green, A Guest, A Harvey, P Kilbourn, A Kucukmetin, M Meirovitz, J Monaghan, R Rangar, N Rashid, A Richardson, B Sarker, M Sihkanyisiwe, A Tailor, G Thompson, G Wilson, B Wright, C Youlton, J Youlton.

Department of Gynaecological Oncology, Nottingham City Hospital, Nottingham (n=16770)
K Williamson (Lead), E Bailey, V Barker, J Barkes, C Bower-Smith, A Bowley, C Bown, S Chowdhary, C Church, V Clements, S Colbeck, F Dack, B Gibbs, M Gill, V Hessom, C Hewitt, R Hutchinson, C Hynes, J Kythreotis, L Lacy, M Mahal, K Manderson, E Mercer, C Norris, D Nunns, C Oakley, T Parkes, C Reynolds, R Rock, H Rushbrook, C Sampson, K Sihra, S Sinclair, Z Thomas, S Thompson, S Vimplis, H Ward, N Ward, K Warner, G Wilson.

Department of Obstetrics and Gynaecology, University of Wales College of Medicine, Cardiff (n=16758)
N Amso (Lead), S Basu, D Bell, H Clarke, A Evans, J Evans, T Griffiths, P Henderson, R Howells, R Jones, G Jose, G Looker, C Morgan, G Rieck, A Rogers, A Sharma, A Sims, S Underwood, D Williams, G Williams.

Department of Gynaecological Oncology, Royal Free Hospital, London (n=16693)

T Mould (Lead), I Aitken, S Amin, I Beal, S Bhola, S Blackmoore, K Borroughs, H Brown, S Burke , D Colia, L Crosby, G Desai, C Efueye, H Evans, E Ferrier, K Fitzgerald, G Fletcher, C Fox, G Gaston, K Harvey, B Heyer, T Hitchen, K Isherwood, E Izard, E Koo, M Lagos, K Lakhani, AM Mackinson, L McKenzie, V Medic, S Mohamed, S More, K Muir, W Myburg, N Nayak, O Ojo, N Old, A Oldham, S Porcherot, E Rawstron, M Sharma, T Stevens, C Sundstrom, E Sweeney, J Terwin, D Townshend, F Turner, S Wellington, D White, J Wickes, L Young, E Zard.

Department of Gynaecological Oncology, St. Michael's Hospital, Bristol (n=16574)
J Murdoch (Lead), B Anderson, F Anderson, H Andrews, E Barrow, R Brown, J Chippett, A D'Angelo, K Gale, N Hammadieh, K Henson, A Hobbs, K Horton-Fawkes, K Hunstman, T James, N Jeal, S King, E Langdon, M Lord, J Marsden-Williams, K McMillan, V Mitchell, K Nicholls, D Park, R Phillips, C Pretsell, RC Sanders, B Schaefer, C Shahin, S Shahin, S Sizer, P Taylor, M Tovey, N Vickers, S Wilmot.

Academic Unit of Obstetrics and Gynaecology, St. Mary's Hospital, Manchester (n=16503)
MW Seif (Lead), K Reynolds (Lead), G Atanga, S Atkinson, L Bailey, C Barber, P Bhakar, N Bhandari, A Blackman, K Bowden, S Briggs, J Brown, D Bushell, K Butler, S Charles, J Collins, M Condon, M Dale, M Doyle, J Dunscombe, R Elfin, R Elven, M Faheem-Siddiqui, MR Green, Z Griffiths, J Harris, N Harwood, J Hawnaur, H Haydock, S Heywood, P Hughes, L Ivers, S Kaye, C Kilkelly, J Lees, M Maheem, G Martin, S Mawn, S McDonald, M Moore, T Morgan, J Nelson, E Oughton, A Panteli, V Parker, J Peacock, C Philipps, J Prior, V Purnell, S Renshaw, L Roberts, S Robin, J Robinson, R Simpson, T Speakman, F Storton, S Subin, J Taylor, X Vanakara, A Webb, C Webb, C Wilde, M Williams, V Williams, A Wood, C Wood, H Wright.

Department of Gynaecological Oncology, Derby City Hospital, Derby (n=14923)
H Jenkins (Lead), I Scott (Lead)A Bali, J Barke, C Benson, C Bower-Smith, H Bullock, J Caborn, S Crockett, A Ferguson, G Forbes, J Gomes, R Harrison, C Hollins, M Jones, A North, R Rock, M Scott, H Stanton, S Thompson, M Tudge, G Turner, J Weston, C Williams.

Department of Gynaecological Oncology, Llandudno Hospital, Llandudno, North Wales (n=14308)
S Leeson (Lead), A Baker, V Byrne, C Chapman, B Davies, S Edwards, J Galley, L Griffiths, S Hogg, JY Houghton-Wright, M Howarth, K Hughes, M Hughes, H Jones, D Longley, A Roberts, L Sharpe, S Thomas, BJ Turner, B Warmington, B Waterson.

Department of Gynaecological Oncology, Belfast City Hospital, Belfast (n=13575)
S Dobbs (Lead), M Alkalbani, M Carey, J Caruth, M Clarke, J Forbes, M Gallagher, B Gibbs, M Hendron, R McClelland, M McComskey, D Morgan, M Murray, A O'Donnell, C Poh, J Price, H Sheldon, M Yoong, A Zawislak.

Department of Gynaecology, Liverpool Women's Hospital, Liverpool (n=10110)
J Herod (Lead), C Atherton, T Aust, L Bailey, S Bassi, L Baty, M Brown, H Burgin, J Carter, J Chapman, B Cheetham, JP Conway, H Crocker, B Daniels, L Diment, A Drought, C Finnegan, K Ford, L Greenfield, S Hailward, J Hazelton, M Herod, S Inwood, S Jones, V Jones, L Korb, H Lee, L Limbert, K Lord, J Maloney, M Maraj, J McCarthy, L McGlynn, D Ndlela, J Newman, A Nicolson, K Pearson, S Pennington, D Petter, P Stewart, A Tannock, B Thomson, A Webster, J Webster, S West, H Wright, G Zabroski.

<u>Department of Gynaecological Oncology, James Cook University Hospital, Middlesbrough (n=9922)</u>

D Cruickshank (Lead), A Bullen, V Chadwick, K Chapman, J Francis, R Goldie, C Ikwan-McCabe, K Jan, D Khan, L Lewis, J Nevin, L Prentis, J Proll, R Shanbhag, G Tarry.
